# The Fate of Bryophyte Sporophytes—Phenology and Vectors of *Buxbaumia viridis* in the Kalkalpen National Park, Austria

**DOI:** 10.3390/plants9101320

**Published:** 2020-10-06

**Authors:** Michaela Kropik, Harald G. Zechmeister, Christian Fuxjäger

**Affiliations:** 1 Institute of Botany, University of Natural Resources and Life Sciences, 1180 Vienna, Austria; michaela.kropik@univie.ac.at; 2 Department of Botany and Biodiversity Research, University of Vienna, 1030 Vienna, Austria; 3 Nationalpark O.ö. Kalkalpen GmbH, Molln, 4591 Kirchdorf an der Krems, Austria; christian.fuxjaeger@kalkalpen.at

**Keywords:** herbivory, mosses, monitoring, deadwood, wildlife camera

## Abstract

Knowledge about the epixylic moss *Buxbaumia viridis* has increased significantly due to the monitoring obligation under the Habitats Directive. However, there are still open questions about its dispersal, as the wind plays a limited role in forest ecosystems, and vectors have been suspected but not yet studied systematically for this species. Here, we present data on potential vectors of *Buxbaumia viridis* collected for the first time with the help of cameras, completed by monitoring the fate of sporophytes during their life cycle in the Limestone Alps National Park in Austria over a period of two years. Young, green sporophytes appeared mainly in autumn, with the highest number in October. Most of them survived winter and spring but did not exceed the age of 14 months. The sharpest decline in capsules occurred in summer when mature, and the lowest number of sporophytes appeared at the end of August. Most likely, mice seem to be responsible for this loss, as the photos from the wildlife cameras suggest, and should be considered both as predators and vectors. Birds should be considered as vectors, too. In summary, most of the reproductive biomass is sacrificed in favor of more effective dispersal, including over longer distances.

## 1. Introduction

*Buxbaumia viridis* is a species of special concern, as it is listed in Annex II of the Habitats Directive. It is distributed all over Europe, and according to the monitoring process demanded by Article 11 of the Habitats Directive, an increasing number of sites have been identified in the last 10 years [1].

As a result, *Buxbaumia viridis* has received increasing attention, and several studies have focused on its biology. Studies on the life cycle of *Buxbaumia viridis* were conducted by Wolf [2] and Callaghan and Taylor [3]. They have described the various life stages within a life cycle, from the first development of a sporophyte to its decay. However, these studies were not based on the observation of a single sporophyte, but characteristics were illustrated by observing different sporophytes at different sites.

These studies, and those of Infante and Heras [4] and Ruete et al. [5], show the high variability of *Buxbaumia viridis*, e.g., in terms of fruiting periods under various climatic conditions. The distribution of *Buxbaumia viridis* in Europe is scattered, as the species has a comparatively narrow niche in terms of substrate and climate. These substrate preferences are documented by a few studies, as follows [6,7]: *Buxbaumia viridis* grows mainly on the dead logs of spruce [8,9], however dead logs of other species (e.g., larch, beech, ash) [4,10,11], as well as the ground [12], can be suitable substrates too. The degree of decay seems to be important, too [13], although the species shows considerable variation in this respect [14]. Regarding the climate, the species prefers high humidity [15,16], however seasonal variations in temperature seem to be important [17]. Furthermore, the relevance of the disposable amounts of deadwood and patch size [15], as well as habitat persistence [10], are emphasized.

Habitat persistence and subsequently habitat availability are key factors for metapopulation dynamics, which seem to be crucial for understanding the distribution and survival of many bryophytes [18], and particularly for *Buxbaumia viridis*, which relies on deadwood—usually rare and nested, and often removed in managed forests. The connectivity of available substrate seems to be important [19], and dispersal within available substrates seems to be a prerequisite for the presence of *Buxbaumia viridis*. Wiklund [15] reports that sites within an adequate microclimate with an adequate amount of deadwood were not occupied. This phenomenon could also be observed within the Kalkalpen National Park [20], which suggests the stronger limitation effect of dispersal compared to habitat availability.

However, the dispersal of *Buxbaumia viridis* is partly still a mystery; it can be by spores and by vegetative propagules (gemmae on the rhizoids). The range of spores is reported as a matter of spore size [18] and suitable vectors [21]; *Buxbaumia viridis* has small spores in enormous numbers (4–6 × 10^6^ on average) [8,15], which generally fosters dispersal by the wind. Further, raindrops were considered to emit puffs of spores when striking the capsule [22]. Commonly, the overall number of bryophyte spores is distributed within a few meters [23], and especially in wood ecosystems the distance is limited due to missing updrafts [24,25]. Furthermore, the life span of dried-out spores is short [7]. It is therefore still unclear how the spores of *Buxbaumia viridis* could reach suitable habitats outside their distribution radius, which is less than a few meters [15,18].

Data on other vectors than the wind are very rare in general for bryophyte spores [26,27], and they are completely missing for *Buxbaumia viridis*. Animals should be considered. Studies on the epizoochory of bryophytes are scarce [26]. Most studies on the endozoochory of bryophyte spores so far focused on slugs [28,29]. Slugs have been reported to damage the capsules of *Buxbaumia viridis* [4,30]. To date, other animals have also been considered [4], however there is no evidence for that. Since the translocation of building material from the gametophyte to the sporophyte during maturation [31] might attract predators, *Buxbaumia viridis*, with its large capsule and high numbers of spores, could be particularly interesting. It has yet to be clarified if the observed losses of capsules of *Buxbaumia viridis* result from predation, and if there are animal vectors fostering dispersal.

Therefore, this study aims to detect potential vectors and predators of spores by monitoring sporophytes of *Buxbaumia viridis* with the use of cameras. Additionally, monitoring at several sites over a period of two years should give further insights into the fate of single sporophytes during their life cycle, and into the number of sporophytes being lost.

## 2. Results

### 2.1. Monitoring of Single Sporophytes Throughout Their Life Cycle

**Figure 1:**
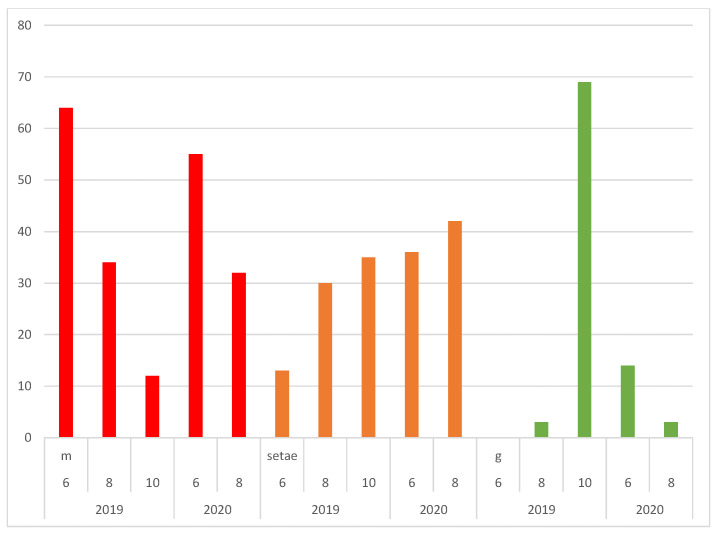
The total number of sporophytes and their variation at five dates of monitoring between mid-June 2019 and the end of August 2020; m—mature sporophytes (red color), setae—seta lacking a capsule (orange), g—green sporophytes (green).

In the monitoring periods 2019/2020, a total of 150 sporophytes from eight clusters of sporophytes (Table 1) was observed. Sporophyte numbers ranged between 2 and 48 on a single log in the year 2019, and between 0 and 69 on a single log in the year 2020 (Figure 1).

The growth season with the highest sporophyte numbers in the area was autumn, and a continuous decrease in sporophytes was observed during summer (Figure 1). Most sporophytes (81 in 2019) were present in October, an average number were present in June (64 sporophytes in 2019, 69 in 2020) and the lowest number were present by the end of August (37 sporophytes in 2019, 35 in 2020). Only several new sporophytes appeared in June, and almost none appeared in August.

In summary, none of the observed sporophytes became older than 14 months, and 80% of the new, green sporophytes from October survived winter and spring. In total, 80% of these were lost when mature during summer, especially in August—55% remained as setae, 25% disappeared completely. Setae in most cases survived for longer than one year.

After the second summer, four out of eight clusters of sporophytes had completely vanished (see Table 2). All clusters at decay stage 4 were lost (Table 1: cluster numbers 1, 2, 7 and 8). At site 1, though, a new large cluster of sporophytes was discovered on a neighboring log instead.

### 2.2. Observations by Cameras

During both recording periods, 141 animals were captured by the wildlife cameras (see Table 3). In total, 65% of these were mammals, 22% were birds, 13% were arthropods and 1% were slugs (for details see Table 3). A small number of releases (<5%) could not be attributed to any animal. Regarding the total number of observations made by cameras, there was no significant difference between the two sites (D(17) = 0.12, *p* = 0.10) and no significant difference between both recording periods (D(17) = 0.29, *p* = 0.45). However, there was a considerable difference between the observation periods regarding the numbers of mammals, arthropods and birds, the latter two dominating in period two, whereas mammals dominated in period one. This is mainly attributed to a marked decrease in mice, which were the overall dominant group at both sites in period one, and to a marked increase in birds at cluster 5 (Table 3).

Among mammals, the main group was mice, mainly consisting of the yellow-necked mouse (*Apodemus flavicollis*) and bank vole (*Myodes glareolus*). Several mice could be seen not only running over the observed clusters of sporophytes, but also stopping and eating sporophytes. A wild boar *(Sus scrofa)* was photographed as it unintentionally trampled a part of the cluster while running over the observed log.

Among birds, song thrushes (*Turdus philomelos*) and robins (*Erithacus rubecula*) were slightly dominant, however other bird species did not markedly differ in number from these (Table 3). All observed birds preferred the ground in the vicinity of the clusters for picking food.

## 3. Discussion

### 3.1. Monitoring of Clusters of Sporophytes

In total, the number of observed sporophytes in June and August was larger in 2020 than in 2019, although four clusters of sporophytes had completely vanished in 2020. The higher number of sporophytes in 2020 can be attributed to the high precipitation in the vegetation period 2020. In contrast to the very dry summers of 2018 and 2019, there was 25% more rain in 2020 than in the long-term average of an Austria-wide analysis [33].

The trigger for the growth of sporophytes in October remains speculative, which is also reported by others [3,8]. Hancock and Brassard [34] and van der Kolk [35] report the beginning of the growing season in September/October and a continuous maturation leading to spore dispersal in June for *Buxbaumia aphylla*, which might be comparable to *Buxbaumia viridis* in some respects. Despite the contrasting climates in the areas of the aforementioned studies (the Netherlands, Newfoundland) and our mountainous study area, the growth and maturation of sporophytes seem to be fairly comparable. However, in contrast to Plášek [8], the sporophytes in our study did not vanish during the wintertime, which could be deduced from the tracking of single sporophytes. The survival until spring, which is very late in the monitoring area, could be attributed to a long-lasting snow cover in the monitoring area, which protects capsules from heavy frost [34] and predators. As found by Hancock and Brassard [34] for *Buxbaumia aphylla*, sporophytes survived wintertime in an immature stage; in the following period, there was a continuous decrease in sporophytes, mainly during the summer period between mid-June and the end of August. This is consistent with other studies on *Buxbaumia viridis* [8] and with our observations.

The fact that in 80% of all cases only setae were left, and capsules were gone, raises the question of the cause. The high number of sporophytes ending up as setae is also reported by Callaghan and Taylor [3]. It is very likely that these capsules were eaten by predators, as not a single broken off capsule was found in the surroundings of the monitoring sites.

### 3.2. Vectors and Predators

The use of cameras for observing clusters of *Buxbaumia viridis* sporophytes showed surprising results (Table 2), especially the large number of mice, small birds and spiders which could be seen around the two observed clusters was striking.

In our study, a large number of sporophytes was lost during the life cycle. Herbivory of sporophytes should be considered as a possible reason. In several studies, gastropods are reported to be the main predators of bryophyte sporophytes [30,36,37]; slugs especially are highlighted, e.g., in [28,29]. In contrast, we hardly found any slugs in our observations. Due to the type of camera, they cannot trigger it. However, if they were to occur frequently in the vicinity of the clusters, a higher number of random shots would seem very likely. Although we searched every picture for slugs in detail, we only found one slug in 1 of 141 pictures. As spiders, which are even smaller than most slugs, could be easily identified on the pictures, it seems very unlikely that we overlooked slugs on the pictures taken. Furthermore, we did not see a single gastropod on *Buxbaumia viridis* during fieldwork in an observation period of four years at more than 100 sites [20,38]. Bryologists so far have not seen most of the other animals captured by the camera, as wildlife is shy and escapes before being detected by a human being.

In our study, a large number of mice was captured in the close vicinity of the sporophytes. Mice should therefore be considered as potential predators of *Buxbaumia viridis*. Sporophytes, rich in protein and lipids, are attractive for small mammals and birds, not only in polar regions [39]. Mature sporophytes appear to be particularly attractive; notably, Rydgren and Økland [31] have shown for *Hylocomium splendens* that the late phase of sporophyte development is the most expensive in terms of resource requirements, because building materials are shifted from the gametophyte to the sporophyte. This might be considered as a possible reason for the higher number of mice recordings in recording period one, in which mature, and thus more energy-rich, sporophytes dominated. The predation of *Buxbaumia viridis* sporophytes by mice is also corroborated by our observations in the virgin forest Rothwald. Compared to the year 2011, hardly any *Buxbaumia viridis* sporophytes could be found in 2012—a year with an enormous mouse population [40]. However, there is some possibility that the higher observation rate during the late phase of sporophyte development is a coincidence, due to varying patterns in the activity of mice [41].

Whether the predation of *Buxbaumia viridis* spores fosters dispersal needs to be clarified. Herbivory seems to be an effective means of dispersal for bryophytes [2,29]. It has been shown that bryophyte spores survive the digestive tract of slugs [28], and that endozoochory by slugs can increase bryophyte establishment [29]. Due to the small home-range of slugs (e.g., 45.2 m^2^ for *Arion lusitanicus* [42]), dispersal is likely to be limited to the short-range.

Vectors with a larger action radius are mice, e.g., 0.37–3.5 ha for *A. flavicollis* and 0.03–0.2 ha for *M. glareolus* [43]. We observed a high number of mice, which should be considered as important vectors in the medium range. The action radius of the observed birds and large mammals in our study, e.g., wild boar, is even larger [44], and these should be considered as potential vectors of *Buxbaumia viridis* in the long range. Furthermore, Heinken et al. [27] proposed the epizoochorous dispersal of bryophyte fragments by roe deer and wild boar as an important vector for bryophytes. Epizoochory might also increase the specificity of dispersal to appropriate biological niches, as animal vectors are likely to be drawn to specific locations within a habitat [45].

The epizoochory of bryophyte propagules by birds has been convincingly presented by Chmielewski and Eppley [26]. They suggest the spread of bryophytes both locally and over long distances by birds, based on the observation of bryophyte spores on tail feathers and legs. Thrushes—one of the dominant birds at our sites—had high numbers of spores on their tail feathers and should therefore be considered as vectors.

In general, the spread of spores by birds is suggested to be a by-product of animal behavior, rather than the result of close co-evolution [26]. Our observations are consistent with this hypothesis, as birds searched the log or the soil around the clusters of sporophytes, but they did not specifically respond to the sporophytes. The peak in bird observations during September/October in our study could be due to the life cycle of songbirds. With the end of the breeding season between June and August, the territorial system of songbirds dissolves, allowing for increased roaming by individuals. At the same time, the new juveniles start to fly, and the total bird population increases by at least a factor of two [46].

In summary, dispersal by animals seems to be more important for *Buxbaumia viridis* than previously thought. The presence of the species in the Kalkalpen National Park is patchy, although existing habitat is certainly not a restriction. For more than 20 years, there has been hardly any forestry intervention in an area of around 200 km^2^. A sufficient amount of deadwood is present in all decay stages and under various microclimatic conditions. Nevertheless, suitable habitats are not occupied, which suggests that *Buxbaumia viridis* in the area is dispersal-limited rather than habitat-limited.

This study provides insights into potential vectors and predators of *Buxbaumia viridis* with the use of cameras for the first time. The greatest loss of sporophytes was observed in the mature state during the summer. The images recorded suggest that mice should be considered both as vectors and predators, whereas birds are more likely vectors by chance. It seems likely that, in favor of more effective dispersal, even over longer distances, most of the reproductive biomass is sacrificed.

## 4. Materials and Methods 

### 4.1. Monitoring Area and Study Sites

The study took place in the Kalkalpen National Park, situated in the Northern Limestone Alps in Upper Austria. The monitoring area is influenced by comparably high precipitation (around 1800 mm/year) and a mean annual temperature of around 6 °C at the altitude of the observed sites. The National Park is dominated by old broadleaved (beech) and mixed forests, as well as by some larch forests at higher altitudes, situated on steep slopes. Deep gorges formed by fast-running streams and several mountain peaks above the timberline constitute the scenery. The monitoring of the clusters of sporophytes took place at six sites—five mixed forests (Site No. 2, 3, 4 and 6 in Table 1) and one spruce forest (Site No. 5).

The selection of sites is based on knowledge from a previous study [20] in the area. Six clusters of sporophytes under favorable conditions—obviously not limited by habitat or climate—were selected. The sites are characterized by a large amount of deadwood and by the fact that they have not been managed for at least 20 years. Two sites (Site No. 1 and 6 in Table 1) are suspected to be virgin forest sites. In any case, it is certain that no human intervention (e.g., logging, grazing) has taken place there for more than 300 years. The altitude of the sites ranges between 1090 m a.s.l. and 1220 m a.s.l. The log diameters were between 25 cm and 50 cm, and the length was between 0.5 m and 20 m (see Table 1). The scale of the decaying stage follows Lachat et al. [32], and was between 2 and 4. At sites 1 and 4, two logs each were monitored, while on all other sites a single log was monitored. Therefore, a total of eight clusters of sporophytes was monitored.

### 4.2. Monitoring of Clusters of Sporophytes

As the gametophyte of *Buxbaumia viridis* is hardly visible, only sporophytes of the species were monitored.

At all selected sites, clusters of *Buxbaumia viridis* sporophytes were regularly monitored between mid-June 2019 and the end of August 2020. All sites were visited three times in 2019 (June, August, October) and two times in 2020 (June, August).

The selected clusters were monitored by a) drawing the positions of each sporophyte at scales of 1:2 and 1:10, respectively, on paper and b) by taking photographs. Additionally, nails were fixed at single sporophytes or between major agglomerations of sporophytes. For the easier relocation of each sporophyte, the distances between these nails were measured. Therefore, it was possible to trace every single sporophyte in the subsequent monitoring periods. The life cycle stages of each sporophyte were monitored too (Table 2).

Monitoring dates were chosen according to experiences gathered via a previous study in the area [20], and due to accessibility regarding snow cover. As determined from this previous study, the period between June and October seemed to be the main growth season of *Buxbaumia viridis* sporophytes in the area.

### 4.3. Recording of Potential Vectors and Predators

Two cameras (Type Bushnell, Modell 119776) were adapted and mounted at two sites (Clu. No. 3 and 5 in Table 1) with large abundances of *Buxbaumia viridis* in 2019. The cameras were mounted at a height of 50 cm above the ground, and at a distance of about 1.5 m from the clusters of sporophytes, to ensure sufficient detail in the recordings. The cameras were triggered by a passive infrared sensor, which registers moving homoiothermal animals in the field of view of the camera in a sector of about 1.5 m radius and with an angle of 90°. After triggering, the observed area was recorded with a short film for 15 s. In addition, a picture was taken. This additional image did not work well and was stopped after the first period, also to reduce data storage. The cameras also worked during the night without any time limit.

At cluster 5, the camera was in operation from 3 August until 9 September 2019, and from 15 September to 17 October 2019. At cluster 3, the camera was in operation from 3 August until 15 September 2019. There was no possibility of a second observation period at site 2 for technical reasons. Recording period one was chosen according to the maturation of sporophytes in that period, and period two according to the presence of fresh green sporophytes in this time.

### 4.4. Statistics

We compared the numbers of observations made by cameras between cluster 3 and cluster 5, as well as between recording period one (03.08.2019–09.09.2019) and recording period two (15.09.2019–17.10.2019), by a Kolmogorov-Smirnov Z Test, using R 3.6.2 (R Foundation for Statistical Computing: Vienna, Austria) [47]. The level of significance was 0.05.

## Figures and Tables

**Table 1:** Investigated sites at the Kalkalpen National Park, Austria; Clu. No.—number of the cluster of sporophytes; decay stage following Lachat, et al. [32].

Site No.	Clu. No.	Site Name	Forest Type	Altitude (m. a.s.l.)	Tree Species	Length of log (m)	Diameter (cm)	Decay Stage
**1**	1	Geißlucke	mixed	1220	beech	20	50	4
**1**	2	Geißlucke	mixed	1220	beech	20	50	4
**2**	3	Groisn ditch	mixed	1160	spruce	3	25	3
**3**	4	Groisn slope	mixed	1170	larch	14	35	2
**4**	5	Groisn plateau	mixed	1190	spruce	4	30	3
**4**	6	Groisn plateau	mixed	1190	larch	4	30	3
**5**	7	Kreuzau	spruce	1090	spruce	0.4	45	4
**6**	8	Trämpl	mixed	1120	spruce	20	40	4

**Table 2:** The number of sporophytes within each cluster and their variation at five monitoring dates between mid-June 2019 and the end of August 2020; Site No.—site number (see Table 1); Clu. No.—number of the cluster of sporophytes; m—mature sporophyte; setae—sporophyte lacking a capsule; g—green sporophyte.

Site No.	Clu. No.	2019_06			2019_08			2019_10			2020_06			2020_08		
		m	setae	g	m	setae	g	m	setae	g	m	setae	g	m	setae	g
**1**	1	13	5	0	9	9	1	6	11	10	3	13	1	0	14	0
**1**	2	5	0	0	1	2	0	0	3	0	0	0	0	0	0	0
**2**	3	14	0	0	8	5	2	1	4	0	0	2	9	5	1	0
**3**	4	4	2	0	2	2	0	2	3	5	5	4	0	4	4	3
**4**	5	17	5	0	10	10	0	2	14	31	26	15	2	3	20	0
**4**	6	4	0	0	1	0	0	1	0	23	21	2	0	20	3	0
**5**	7	5	1	0	2	2	0	0	0	0	0	0	2	0	0	0
**6**	8	2	0	0	1	0	0	0	0	0	0	0	0	0	0	0
**sum**		64	13	0	34	30	3	12	35	69	55	36	14	32	42	3

**Table 3:** Type and number of animals captured by the automatic release of cameras at two sites within the respective months of observation; Clu. No.—cluster number (see Table 1); Series 1: 3 August–9 September 2019, Series 2: 15 September–17 October 2019; indet. = indeterminable.

Classes	Animals	Clu. No. 3	Clu. No. 5		Hits In Total	% of Total Hits
		Series 1	Series 1	Series 2		
mammals		41	36	14	91	65
birds		2	3	26	31	22
arthropods		1	4	13	18	13
gastropods				1	1	1
total number					141	
	badgers			1	1	0.7
	squirrels		3		3	2.1
	deer			4	4	2.8
	martens	4	2		6	4.3
	mice	35	30	9	74	52.5
	humans		1		1	0.7
	roe deer	1			1	0.7
	boars	1			1	0.7
	blackbirds			5	5	3.5
	capercaillies	2	1		3	2.1
	song thrushes		2	7	9	6.4
	robins			9	9	6.4
	birds indet.			2	2	1.4
	wrens			3	3	2.1
	spiders	1	3	13	17	12.1
	ground beetles		1		1	0.7
	slugs			1	1	0.7
	sum	44	43	54	141	100
